# LINC00978 promotes the progression of hepatocellular carcinoma by regulating EZH2-mediated silencing of p21 and E-cadherin expression

**DOI:** 10.1038/s41419-019-1990-6

**Published:** 2019-10-03

**Authors:** Xueying Xu, Jianmei Gu, Xiaoge Ding, Guohong Ge, Xueyan Zang, Runbi Ji, Meng Shao, Zheying Mao, Yu Zhang, Jiayin Zhang, Fei Mao, Hui Qian, Wenrong Xu, Hui Cai, Feng Wang, Xu Zhang

**Affiliations:** 1 Jiangsu Key Laboratory of Medical Science and Laboratory Medicine, School of Medicine, Jiangsu University, 301 Xuefu Road, Zhenjiang, Jiangsu 212013 China; 2 Departmemt of Clinical Laboratory Medicine, Nantong Tumor Hospital, 30 Tongyang North Road, Nantong, Jiangsu 226361 China; 3 Liver Disease and Cancer Institute, The Affiliated Zhenjiang Third Hospital of Jiangsu University, 300 Daijiamen Road, Zhenjiang, Jiangsu 212021 China; 4 Key Laboratory of Molecular Diagnostics and Precision Medicine for Surgical Oncology in Gansu Province, Gansu Provincial Hospital, 24 West Donggang Road, Gansu, 730000 China; 5 Departmemt of Clinical Laboratory Medicine, Affiliated Hospital of Nantong University, 20 Xisi Road, Nantong, Jiangsu 226001 China

**Keywords:** Long non-coding RNAs, Diagnostic markers

## Abstract

Long non-coding RNAs (lncRNAs) have been suggested as important regulators of cancer development and progression in hepatocellular carcinoma (HCC). Nevertheless, the clinical value and biological roles of LINC00978 in HCC remain unclear. In this study, we detected the expression of LINC00978 in tumor tissues and serum of HCC patients, examined the roles of LINC00978 in HCC progression and elucidated the underlying molecular mechanisms. We found that LINC00978 expression was upregulated in tumor tissues and serum of HCC patients. Higher serum levels of LINC00978 could distinguish HCC patients from hepatitis and liver cirrhosis patients and healthy controls. LINC00978 knockdown inhibited HCC cell proliferation, migration and invasion while promoted cell cycle arrest and apoptosis. Overexpression of LINC00978 led to the opposite effects. LINC00978 knockdown also inhibited HCC growth and metastasis in mouse tumor models. Mechanistically, LINC00978 bound to EZH2 and mediated its accumulation at the promoter region of p21 and E-cadherin genes, leading to the trimethylation of H27K3 and the inhibition of p21 and E-cadherin expression. Moreover, the simultaneous depletion of p21 and E-cadherin expression reversed the inhibitory effects of LINC00978 knockdown on HCC cell proliferation, migration, and invasion. Taken together, these findings suggest that LINC00978 promotes HCC progression by inhibiting p21 and E-cadherin expression via EZH2-mediated epigenetic silencing. LINC00978 may represent a novel biomarker for HCC diagnosis, prognosis, and therapy.

## Introduction

Hepatocellular carcinoma (HCC) is one of the most common types of cancers and the third leading cause of cancer-related mortality worldwide^1^. Surgical resection is the best treatment, however, survival rate is still poor in patients with HCC because of the lack of a typical clinical presentation and specific indicator in the early stage. Therefore, it is urgent to discover and develop effective biomarkers and targets for better diagnosis and treatment.

Long non-coding RNAs (lncRNAs) are a class of transcripts that are more than 200 nt in length^2^. They were initially considered as transcription noise due to their lack of protein-coding capacity, but now are thought as important players in human health and diseases. Increasing evidence suggest that lncRNAs are involved in many biological processes of HCC, including initiation, growth, metastasis, and therapy resistance^3–5^. For instance, DANCR and MUF could increase the stemness features of HCC cells^6,7^. LncTCF7 maintains the ability of liver cancer stem cells to self-renew by activating Wnt signaling^8^. HULC is significantly highly expressed in tumor tissues and plasma of patients with HCC and could promote chemoresistance by inducing cell autophagy^9,10^. Lnc-UCID regulates cell cycle that promotes G1/S transition and HCC cell growth^11^. TUG1 is upregulated in HCC and could promote cell growth by inhibiting KLF2^12^. On the contrary, LINC01093, and MIR22HG, two liver-specific long non-coding RNAs, could suppress HCC progression^13,14^. Altogether, these findings suggest that lncRNA is critically involved in the pathogenesis of HCC and may be utilized as biomarkers for HCC diagnosis and prognosis.

LINC00978, also known as MIR4435-2HG and AK001796, is a lncRNA located in the 2q13 region of human genome. LINC00978 upregulation could promote cell proliferation and invasion in non-small cell lung cancer^15,16^. Deng et al. demonstrate that LINC00978 is overexpressed in breast cancer and high level of LINC00978 is associated with poor prognosis^17^. Bu et al. demonstrate that LINC00978 promotes cell proliferation and tumorigenesis via regulating miRNA-497/NTRK3 axis in gastric cancer^18^. Recently, we have also shown that LINC00978 is upregulated in gastric cancer and its expression level is associated with gastric cancer progression^19^. However, the biological roles of LINC00978 in HCC and its potential mechanism of action have not been well characterized.

In this study, we determined the biological function of LINC00978 in HCC progression and investigated the underlying molecular mechanism. Our results showed that LINC00978 was upregulated in tissues and serum of HCC patients. The upregulation of LINC00978 had a high diagnostic value to distinguish HCC patients from patients with hepatitis and liver cirrhosis and healthy controls. LINC00978 promoted HCC cell proliferation and enhanced the metastatic potential of HCC cells in vitro and in vivo. LINC00978 exerted its oncogenic activities through binding with EZH2 to epigenetically silence p21 and E-cadherin expression. Our results suggest that LINC00978 is critically involved in HCC progression and may serve as a potential diagnostic biomarker for HCC.

## Materials and methods

### Microarray data analysis

Human microarray dataset GSE64041 was obtained from the Gene Expression Omnibus (GEO database, http://www.ncbi.nlm.nih.gov/geo/). The GSE64041 dataset contained the transcriptome data of 120 pairs of tumor tissues and parenchyma normal tissues studied on an Affymetrix Human Gene 1.0 ST Array (Affymetrix, Santa Clara, CA, USA), with data normalized by the Robust Multichip Average (RMA) algorithm. The z-score of log2 format of normalized data was used for further analysis.

### Clinical samples and data collection

Clinical samples including tissue and serum samples were collected from the Affiliated Hospital of Jiangsu University between April 2015 and December 2016. All selected hepatocellular carcinoma patients met the following inclusion criteria: (i) Patients were newly diagnosed to have hepatocellular carcinoma with definite pathological evidence or radiological evidence; (ii) No chemoradiotherapies were given before surgery. Preoperative blood samples of hepatocellular carcinoma patients, benign liver disease patients and healthy controls were collected, separated for serum within 2 h and stored at −80 °C. Tissue samples including tumor and paired non-tumor tissues were collected within 1 h after liver resection. Tissue samples were put into liquid nitrogen immediately and then transferred to −80 °C for storage. This study was performed with the approval of Ethics Review Committee of Jiangsu University. Informed consents were obtained from each participants included in the study prior to sample collection. All experiments were performed in accordance with relevant regulations and guidelines.

### Cell culture

The human hepatocellular carcinoma cell lines 7721, 7402, HepG2, and LM3 were obtained from Shanghai Institute of Biochemistry and Cell Biology, Chinese Academy of Sciences (Shanghai, China). The human normal liver epithelial cell line 7702 was purchased from Gefan Biological Technology (Shanghai, China). 7702, 7402, 7721, and HepG2 was cultured with high glucose-Dulbecco’s modified Eagle medium (DMEM; Gibco, Grand Island, NY, USA), while LM3 cells were cultured with Roswell Park Memorial Institute (RPMI) 1640 medium (Invitrogen, Carlsbad, CA, USA). All media were supplemented with 10% fetal bovine serum (FBS; Gibco), 100 U/mL penicillin and 100 μg/mL streptomycin (Gibco). All the cells were cultured at 37 °C in a humidified incubator with 5% CO_2_.

### RNA isolation, reverse transcription, and quantitative real-time polymerase chain reaction (qRT-PCR)

Total RNA from serum samples and serum exosomes was extracted by using miRNeasy Serum/Plasma kit according to the manufacturer’s instructions (QIAGEN, Hilden, Germany). TRIzol reagent (Invitrogen) was used for the extraction of total RNA from the tissue samples and cultured cells. The cDNAs were generated from 1 μg total RNA by using the HiScript 1st Strand cDNA Synthesis Kit (Vazyme, Nanjing, China). QRT-PCR was performed using UltraSYBR Mixture (CWBIO) on a CFX96 Real-time PCR Detection System (Bio-Rad, Hercules, CA, USA). The qRT-PCR reactions were performed in triplicate. Changes in gene expression were determined by the −ΔCt or 2^−ΔΔCt^ method. Results were normalized to the expression of small molecular RNA U6 and β-actin. The sequences of primers used for qRT-PCR were presented in Table 1.

**Table 1:** Primer sequences of target genes

Target	Sequence (5′-3′)	Size (bp)	Tm (°C)
U6	F: 5′-CTCGCTTCGGCAGCACA-3′	94	55
	R: 5′-AACGCTTCACGAATTTGCGT-3′		
LINC00978	F: 5′-AGGCCCCAGGGAATCTTTCA-3′	158	55
	R: 5′-GCCTCTCCCTGAATAACTGGG-3′		
E-cadherin	F: 5′-CGCATTGCCACATACACTCT-3′	252	55
	R: 5′-TTGGCTGAGGATGGTGTAAG-3′		
N-cadherin	F: 5′-AGTCAACTGCAACCGTGTCT-3′	337	55
	R: 5′-AGCGTTCCTGTTCCACTCAT-3′		
Vimentin	F: 5′-GAGCTGCAGGAGCTGAATG-3′	344	55
	R: 5′-AGGTCAAGACGTGCCAGAG-3′		
Slug	F: 5′-CCTGGTTGCTTCAAGGACAC-3′	395	55
	R: 5′-TCCATGCTCTTGCAGCTCTC-3′		
Snail	F: 5′-GCGAGCTGCAGGACTCTAAT-3′	310	55
	R: 5′-GCCTCCAAGGAAGAGACTGA-3′		
Twist	F: 5′-ACGAGCTGGACTCCAAGATG-3′	484	55
	R: 5′-GGCACGACCTCTTGAGAATG-3′		
Bcl-2	F: 5′-GGATCCAGGATAACGGAGGC-3′	150	55
	R: 5′-CCAGATAGGCACCCAGGGT-3′		
Cyclin D1	F: 5′-CCGAGAAGCTGTGCATCTAC-3′	221	55
	R: 5′-CTTCACATCTGTGGCACAGAG-3′		
p21	F: 5′-GAGACTCTCAGGGTCGAAAACG-3′	95	55
	R: 5′-GGATTAGGGCTTCCTCTTGGA-3′		
Actin	F: 5′-CACGAAACTACCTTCAACTCC-3′	265	55
	R: 5′-CATACTCCTGCTTGCTGATC-3′		

### Cell transfection

The overexpressing plasmid and shRNA targeting LINC00978 were synthesized by Hanbio Biotechnology (Shanghai, China). The siRNAs targeting EZH2, p21, and E-cadherin were synthesized by Genepharma (Shanghai, China). Cells cultured in 6-well plate were transfected using LipoFiter (Hanbio, Shanghai, China) according to the manufacturer’s instructions. Cells were changed to complete medium at 6 h after transfection and cultured for another 24 h. The sequences for siRNAs and shRNAs were listed in Table 2.

**Table 2:** Sequences for siRNAs and shRNAs

Target	Sequence
sh-control	5′-CACCGTTCTCCGAACGTGTCACGTCAAGAGATTACGTGACACGTTCGGAGAATTTTTTG-3′
sh-LINC00978	5′-CACCGCCCAGATTTAAGGGCTATTTCAAGAGAATAGCCCTTAAATCTGGGCCTTTTTTG-3′
si-Scr	Sense: 5′-UUCUCCGAACGUGUCACGUTT-3′ Antisense: 5′-ACGUGACACGUUCGGAGAATT-3′
si-p21	Sense: 5′-GGAACAAGGAGUCAGACAUTT-3′ Antisense: 5′-AUGUCUGACUCCUUGUUCCTT-3′
si-E-cadherin	Sense: 5′-CCUGCCAAUCCCGAUGAAATT-3′ Antisense: 5′-UUUCAUCGGGAUUGGCAGGTT-3′
si-EZH2	Sense: 5′-CGGCUUCCCAAUAACAGUATT-3′ Antisense: 5′-UACUGUUAUUGGGAAGCCGTT-3′

### Cell counting and colony formation assay

The transfected cells were seeded into 24-well plates (1 × 10^4^ cells per well). The cells were trypsinized and counted every day for 6 days. The results were plotted as cell growth curves. For colony formation assay, the transfected cells were plated into 6-well plates (1 × 10^3^ cells per well) and cultured for 10 days, during which the medium was replaced every 3 days. At the end of the experiment, the colonies were washed with phosphate buffered saline (PBS), fixed with 4% paraformaldehyde for 30 min and stained with 0.5% crystal violet for 15 min. The number of visible colonies containing ≥ 50 cells was counted. All these experiments were performed in triplicate.

### Flow cytometric analyses of cell cycle and apoptosis

For cell cycle analysis, cells were harvested and fixed with 75% ethanol at 4 °C overnight, then treated with RNase A and stained with propidium iodide (PI) for 30 min at 37 °C. Cell cycle profiles were determined using FACSCalibur flow cytometer (BD Biosciences, San Jose, CA, USA). For the analysis of cell apoptosis, cells were collected and stained with Annexin V-FITC and PI for 15 min in darkness at room temperature. Cells undergoing early and late apoptosis were then quantified using flow cytometry (BD Biosciences). The experiments were performed in triplicate.

### Cell migration and invasion assays

The transfected cells resuspended in 200 μL serum-free media were seeded into the upper chamber of transwell with 8 μm pore (Corning, NY, USA), which was pre-coated with (for invasion assay) or without (for migration assay) 50 μL matrigel. A total of 600 μL DMEM medium or 1640 medium containing 10% FBS was added into the lower chamber. After incubation at 37 °C with 5% CO_2_ for 24 h, cells on the upper surface were removed with a cotton swab and cells on the lower surface were fixed in paraformaldehyde for 30 min followed by staining with 0.2% crystal violet for 15 min. Cells were photographed and counted under an inverted microscope (Olympus, Tokyo, Japan). The results were shown as mean values of three independent experiments.

### Western blot

Cells were washed and lysed in radio-immunoprecipitation assay (RIPA) extraction reagent (Beyotime, Beijing, China) supplemented with a protease inhibitor cocktail solution (Roche, Indianapolis, IN, USA). The protein samples were separated on 10% SDS-polyacrylamide gel electrophoresis and transferred to 0.22 μm PVDF membranes (Millipore, Billerica, MA, USA). After blocking with 5% non-fat milk, the membranes were incubated with specific primary antibodies against p21, Bcl-2, Slug, Snail, Twist, E-cadherin, N-cadherin, Vimentin, and Cyclin D1 (Cell Signaling Technology, Shanghai, China) at 4 °C overnight and washed extensively. After incubation with the goat anti-rabbit or anti-mouse secondary antibodies (Cell Signaling Technology), the protein bands were visualized by using chemiluminescence (Millipore). GAPDH (Sigma-Aldrich, St. Louis, MO, USA) was used as loading control.

### In vivo tumor growth and metastasis studies

All procedures for animal experiments were approved by the Animal Use and Care Committee of Jiangsu University. Ten BALB/c nude mice (4 weeks old, male) were randomly divided into two groups. HepG2 cells transfected with sh-LINC00978 or sh-control were harvested at 24 h after transfection. HepG2 cells (2 × 10^6^) were resuspended in 0.2 mL PBS and injected into the mice subcutaneously. Xenograft tumors were observed periodically after cell inoculation. The mice were killed at 4 weeks after injection and the tumors were removed and photographed. Tumor diameters were examined with a Vernier caliper. Tumor volumes (*V*) were calculated using the formula: *V* = ½ × length × width^2^ (mm^3^). For lung metastasis model, HepG2 cells (2 × 10^6^) were resuspended in 0.2 mL PBS and injected into the mice intravenously. The mice were sacrificed at 6 weeks after injection and the lungs were collected and examined for metastatic nodules.

### Immunohistochemical staining

Tumor tissue sections were incubated with primary monoclonal antibodies against Ki-67, p21, and E-cadherin (Cell Signaling Technology) followed by incubation with the secondary antibody for 30 min at room temperature. After being incubated with 3,3′-Diaminobenzidine (3,3′-DAB, Maxim, Fuzhou, China) for 5 min, the sections were counterstained with hematoxylin for 30 s. Finally, the sections were photographed under a TE2000 microscope (Nikon, Tokyo, Japan). TUNEL (terminal deoxynucleotidyl transferase dUTP nick end labeling) staining was preformed to detect apoptotic cells in tumor tissues following the manufacturer’s instructions (Boster, Wuhan, China).

### RNA immunoprecipitation assay

RNA immunoprecipitation (RIP) assay was performed by using a Magna RIP kit (Millipore) according to the instruction of the manufacturer. Whole-cell lysate was incubated with RIP buffer containing magnetic beads which had been conjugated with human anti-EZH2 antibody, or mouse IgG as negative control. The immunoprecipitated RNAs were extracted, purified, and analyzed by using qRT-PCR to detect the binding of target RNAs.

### Chromatin immunoprecipitation assay

The ChIP assays were performed by using the EZ-ChIP KIT according to the manufacturer’s instructions (Millipore, Billerica, MA, USA). EZH2 and H3K27me3 antibodies were obtained from Abcam (Hercules, CA, USA). Quantification of immunoprecipitated DNA was performed by using UltraSYBR Mixture (CWBIO) on a CFX96 Real-time PCR Detection System (Bio-Rad, Hercules, CA, USA). The sequences of primers used for ChIP-PCR were presented in Table 3.

**Table 3:** The sequences of ChIP primers

Target	Sequence (5′-3′)	Size (bp)	Tm (^o^C)
p21	F: 5′-CTGCCTCTGCTCAATAATGTTCT-3′	85	55
	R: 5′-GGAATTCACCTTCACACAGGC-3′		
E-cadherin	F: 5′-AGACCCCATCTCCAAAACGAACAAA-3′	201	55
	R: 5′-GCATAGACGCGGTGACCCTCTAGCC-3′		

### Statistical analysis

All the data were analyzed by using SPSS 22.0 software (SPSS, Chicago, IL, USA) or GraphPad Prism 7.0 (GraphPad Software, La Jolla, CA, USA). Differences between measured groups were assessed using Student’s *t* test. The associations between LINC00978 expression and clinicopathological features were studied using chi-square test and Fisher’s exact test. The area under the ROC curve (AUC) was analyzed to estimate the effectiveness of LINC00978 for prediction. All *P* values were two-sided. Differences were considered as statistically significant for *P* values < 0.05. Data were presented as mean with the standard deviation (SD).

## Results

### LINC00978 is highly expressed in HCC tissues, serums, and cell lines

**Fig. 1:**
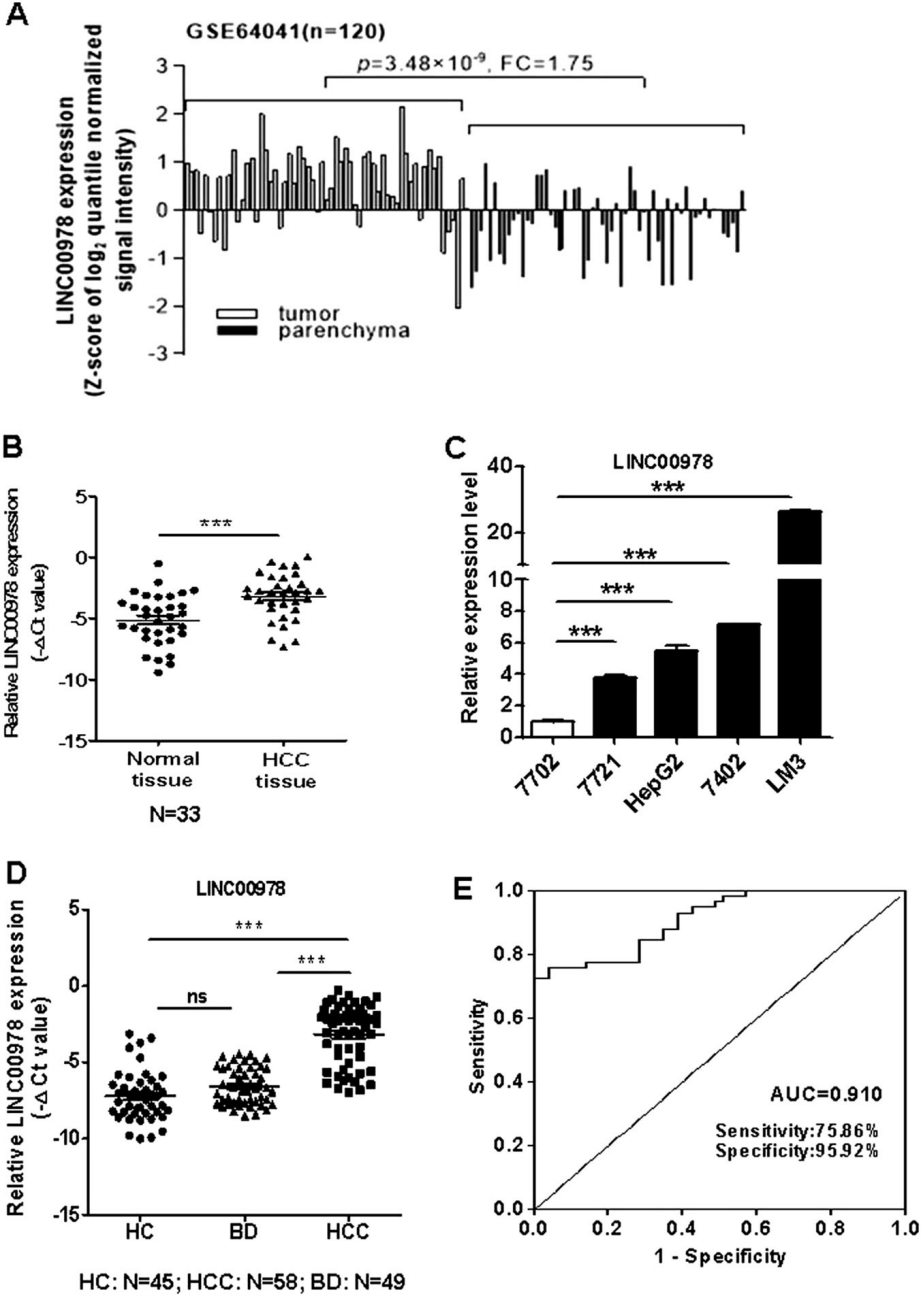
LINC00978 is upregulated in HCC tissues, serums and cell lines. **a** Analysis of LINC00978 expression level in GEO dataset GSE64041. **b** Relative expression of LINC00978 in paired tumor and non-tumor tissues (*n* = 33). **c** The expression profiles of LINC00978 in 7721, HepG2, 7402, LM3, and 7702 cells. **d** LINC00978 expression levels in serum of HCC patients (*n* = 58), liver benign disease patients (*n* = 49) and healthy controls (*n* = 45). **e** ROC curve analysis of the diagnostic performance of serum LINC00978. *P < 0.05, **P < 0.01, ***P < 0.001

We first analyzed the expression levels of LINC00978 in human HCC tissues using the microarray data downloaded from GEO (GSE64041). The results showed that LINC00978 expression level was upregulated in HCC tissues compared with the parenchymal normal tissues (Fig. 1a). To validate the findings of GEO data analysis, we examined LINC00978 expression in a cohort of 33 paired HCC and adjacent non-cancerous tissues by using qRT-PCR. Consistently, the expression level of LINC00978 was also significantly upregulated in HCC issues compared with the paired non-cancerous tissues (*P* < 0.001, Fig. 1b). Moreover, five cell lines including one normal liver epithelial cell line 7702 and 4 HCC cell lines (7721, 7402, HepG2, LM3) were detected for LINC00978 expression. The expression levels of LINC00978 in 7721, 7402, HepG2, and LM3 cells were significantly higher than that in 7702 cells (Fig. 1c). We further tested the expression of LINC00978 in serum samples from HCC patients, liver benign disease patients (hepatitis and liver cirrhosis) and healthy controls. The results showed that the expression levels of serum LINC00978 were significantly higher in HCC patients than those in liver benign disease patients and healthy controls, but the expression levels between liver benign disease patients and healthy controls had no statistical difference (Fig. 1d). The area under the receiver operating characteristic (ROC) curve (AUC) was 0.910 (sensitivity 0.76, specificity 0.96) (Fig. 1e). In summary, these data suggest that LINC00978 is highly expressed in HCC and may serve as a potential diagnostic biomarker.

### LINC00978 knockdown inhibits HCC cell proliferation, migration, and invasion

**Fig. 2:**
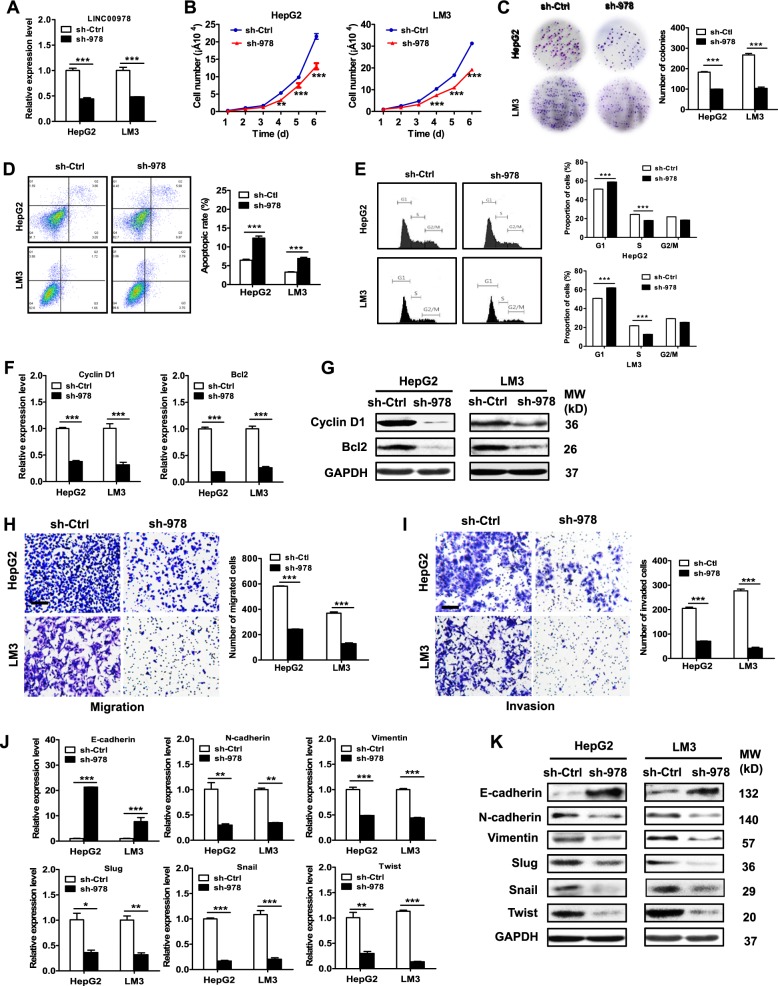
LINC00978 knockdown inhibits proliferation, migration and invasion of HCC cells. **a** Relative expression levels of LINC00978 in shRNA transfected cells. **b** Growth curves of HCC cells transfected with sh-LINC00978 (sh-978). **c** Colony formation assays for HCC cells transfected with sh-LINC00978. **d**, **e** Cell cycle distribution (**d**) and cell apoptosis (**e**) in HepG2 and LM3 cells transfected with sh-LINC00978 were determined by using flow cytometry. **f**, **g** qRT-PCR (**f**) and western blot (**g**) analyses of Cyclin D1 and Bcl-2 expression in HepG2 and LM3 cells with LINC00978 knockdown. **h**, **i** Transwell migration assays (**h**) and Matrigel invasion assays (**i**) were performed to investigate the migratory and invasive abilities of HepG2 and LM3 cells with LINC00978 knockdown. **j**, **k** qRT-PCR (**j**) and western blot (**k**) analyses of EMT-specific markers in HepG2 and LM3 cells with LINC00978 knockdown. Scale bar: 100 μm. *P < 0.05, **P < 0.01, ***P < 0.001

The increased expression of LINC00978 in HCC led us to hypothesize that LINC00978 might function as an oncogene in HCC. To this end, we knocked down LINC00978 expression in HCC cells by using shRNA. The knockdown efficiency was verified by using qRT-PCR (Fig. 2a). The results of cell counting assay showed that LINC00978 knockdown inhibited HCC cell proliferation (Fig. 2b), which was further confirmed by the results of colony formation assays (Fig. 2c). In addition, HCC cells transfected with LINC00978 shRNA had decreased S phase and increased apoptosis (Fig. 2d, e). Moreover, the expression levels of Bcl-2 and Cyclin D1 genes and proteins were decreased in LINC00978 shRNA transfected HCC cells (Fig. 2f, g). The number of migrated and invaded cells was decreased in sh-LINC00978 group compared with that in control group (Fig. 2h, i). The expression of epithelial marker E-cadherin was increased while that of mesenchymal markers N-cadherin and Vimentin was decreased in LINC00978 knockdown cells. In addition, the expression of EMT transcription factors including slug, snail, and twist was significantly downregulated in sh-LINC00978 transfected cells (Fig. 2j, k).

### LINC00978 upregulation promotes HCC cell proliferation, migration, and invasion

**Fig. 3:**
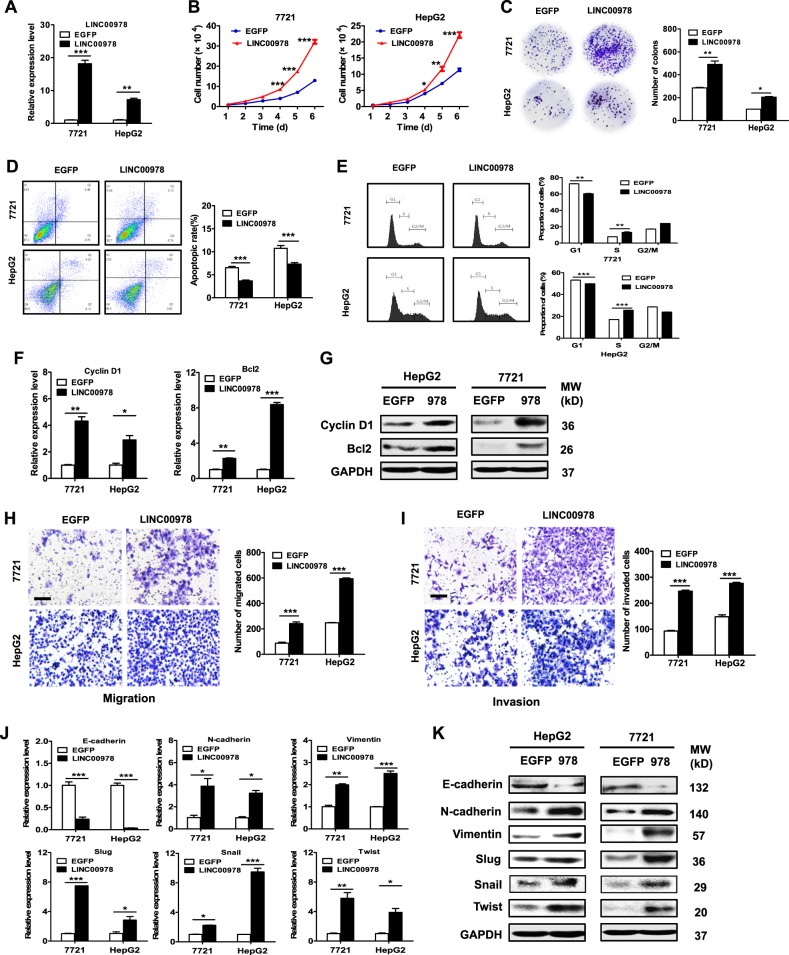
LINC00978 overexpression promotes proliferation and inhibits apoptosis of HCC cells. **a** Relative expression levels of LINC00978 in plasmid transfected cells. **b** Growth curves of HCC cells transfected with LINC00978. **c** Colony formation assays for HCC cells transfected with LINC00978. **d**, **e** Cell cycle distribution (**d**) and cell apoptosis (**e**) in HepG2 and 7721 cells transfected with LINC00978 were determined by using flow cytometry. **f**, **g** qRT-PCR (**f**) and western blot (**g**) analyses of Cyclin D1 and Bcl-2 expression in HepG2 and 7721 cells with LINC00978 overexpression. **h**, **i** Transwell migration assays (**h**) and Matrigel invasion assays (**i**) were performed to investigate the migratory and invasive abilities of HepG2 and 7721 cells with LINC00978 overexpression. **j**, **k** qRT-PCR (**j**) and western blot (**k**) analyses of EMT-specific markers in HepG2 and 7721 cells with LINC00978 overexpression. Scale bar: 100 μm. *P < 0.05, **P < 0.01, ***P < 0.001

We further performed overexpression studies to assess the biological roles of LINC00978 in HCC (Fig. 3a). The results of cell counting and colony formation assays showed that LINC00978 overexpression remarkably promoted the growth of HCC cells (Fig. 3b, c). The percentage of G1 phase and apoptotic cells was decreased in LINC00978 overexpressing HCC cells compared with control cells (Fig. 3d, e). The expression levels of Bcl-2 and Cyclin D1 genes and proteins were increased in LINC00978 overexpressing HCC cells (Fig. 3f, g). In addition, the number of migrated and invaded cells increased in LINC00978 overexpressing HCC cells compared with control cells (Fig. 3h, i). Moreover, the results of qRT-PCR and western blot showed that the expression of E-cadherin decreased while that of N-cadherin, vimentin, slug, snail, and twist increased in HCC cells with LINC00978 overexpression (Fig. 3j, k). These data suggest that LINC00978 overexpression promotes HCC cell proliferation, migration and invasion.

### LINC00978 epigenetically silences p21 and E-cadherin transcription by binding to EZH2

**Fig. 4:**
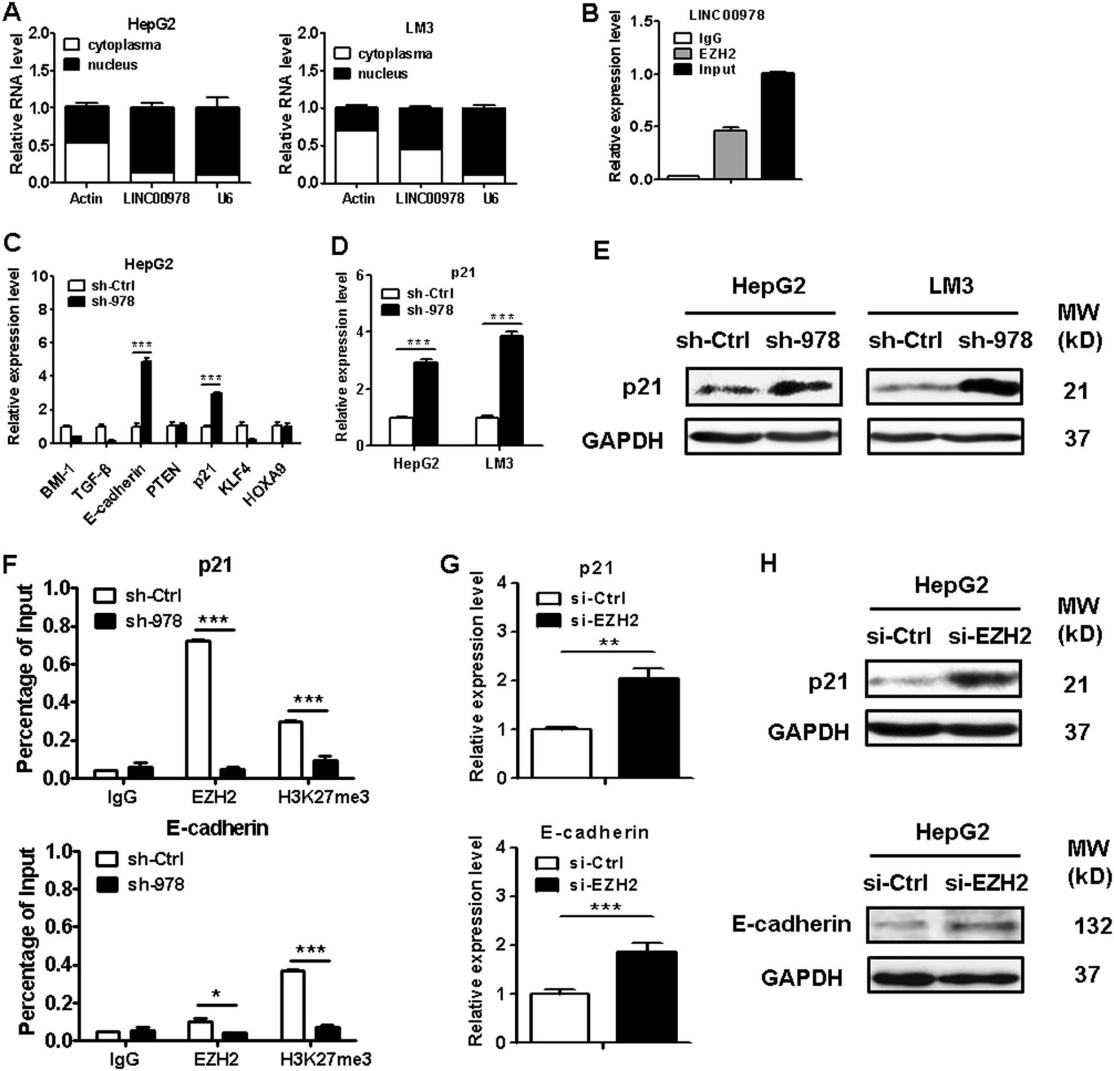
LINC00978 interacts with EZH2 and downregulates p21 and E-cadherin expression in HCC cells. **a** LINC00978 expression levels in the cytoplasm or nucleus of HepG2 and LM3 cells. Actin was used as a cytosol marker and U6 was used as a nuclear marker. **b** RIP assay was performed in HepG2 cells and the co-precipitated RNA was subjected to qRT-PCR for LINC00978. LINC00978 RNA expression levels are presented as fold enrichment in EZH2 immunoprecipitates relative to that of IgG. **c** The expression of potential target genes in LINC00978 knockdown HCC cells was determined by qRT-PCR. **d** The expression of p21 in LINC00978 knockdown HCC cells was determined by qRT-PCR. **e** Western blot analyses of p21 protein expression in HepG2 and LM3 cells transfected with sh-LINC00978. **f** ChIP-PCR of EZH2 occupancy and H3K27me3 binding to p21 and E-cadherin promoters in HepG2 cells with LINC00978 knockdown; IgG as a negative control. **g**, **h** qRT-PCR (**g**) and western blot (**h**) assays were used to detect p21 and E-cadherin mRNA and protein levels in HepG2 cells transfected with si-EZH2. *P < 0.05, **P < 0.01, ***P < 0.001

To explore the molecular mechanisms by which LINC00978 contributes to the malignant behaviors of HCC cells, we detected the subcellular localization of LINC00978 in HCC cells using fractionation assays. LINC00978 expression was higher in the nucleus than in the cytosol in HCC cells (Fig. 4a), indicating that it may function as a regulator of gene transcription. Previous studies have shown that lncRNAs could recruit polycomb repression complex 2 (PRC2) to the promoter of target genes and influence the expression of downstream targets^12^. We chose EZH2, a core subunit of PRC2, to perform RNA immunoprecipitation assays. We confirmed that LINC00978 bound directly to EZH2 in HepG2 cells (Fig. 4b), suggesting that LINC00978 may regulate gene expression by interaction with EZH2. To further investigate the potential target genes involved in LINC00978-meidated HCC progression, we determined the expression of proliferation and EMT related genes that have been verified to be regulated by lncRNA through EZH2 in LINC00978 knockdown HCC cells and chose p21 and E-cadherin as targets for further study (Fig. 4c). qRT-PCR results showed that downregulation of LINC00978 significantly increased p21 and E-cadherin expression (Figs. 3j, 4d). In addition, clear increase in p21 and E-cadherin protein levels was also observed in LINC00978 knockdown HCC cells (Fig. 4e). To further determine whether LINC00978 silenced p21 and E-cadherin transcription by recruiting EZH2 to its promoter, we performed chromatin immunoprecipitation assays in control and LINC00978 knockdown HCC cells. The results showed that EZH2 could bind to the promoter region of p21 and E-cadherin genes, while knockdown of LINC00978 reduced this binding (Fig. 4f). We also found that EZH2 inhibition upregulated the mRNA and protein levels of p21 and E-cadherin (Fig. 4g, h). These data suggest that LINC00978 represses p21 and E-cadherin expression in HCC cells via interaction with EZH2.

### The promotion of HCC cell proliferation, migration, and invasion by LINC00978 is EZH2-dependent

**Fig. 5:**
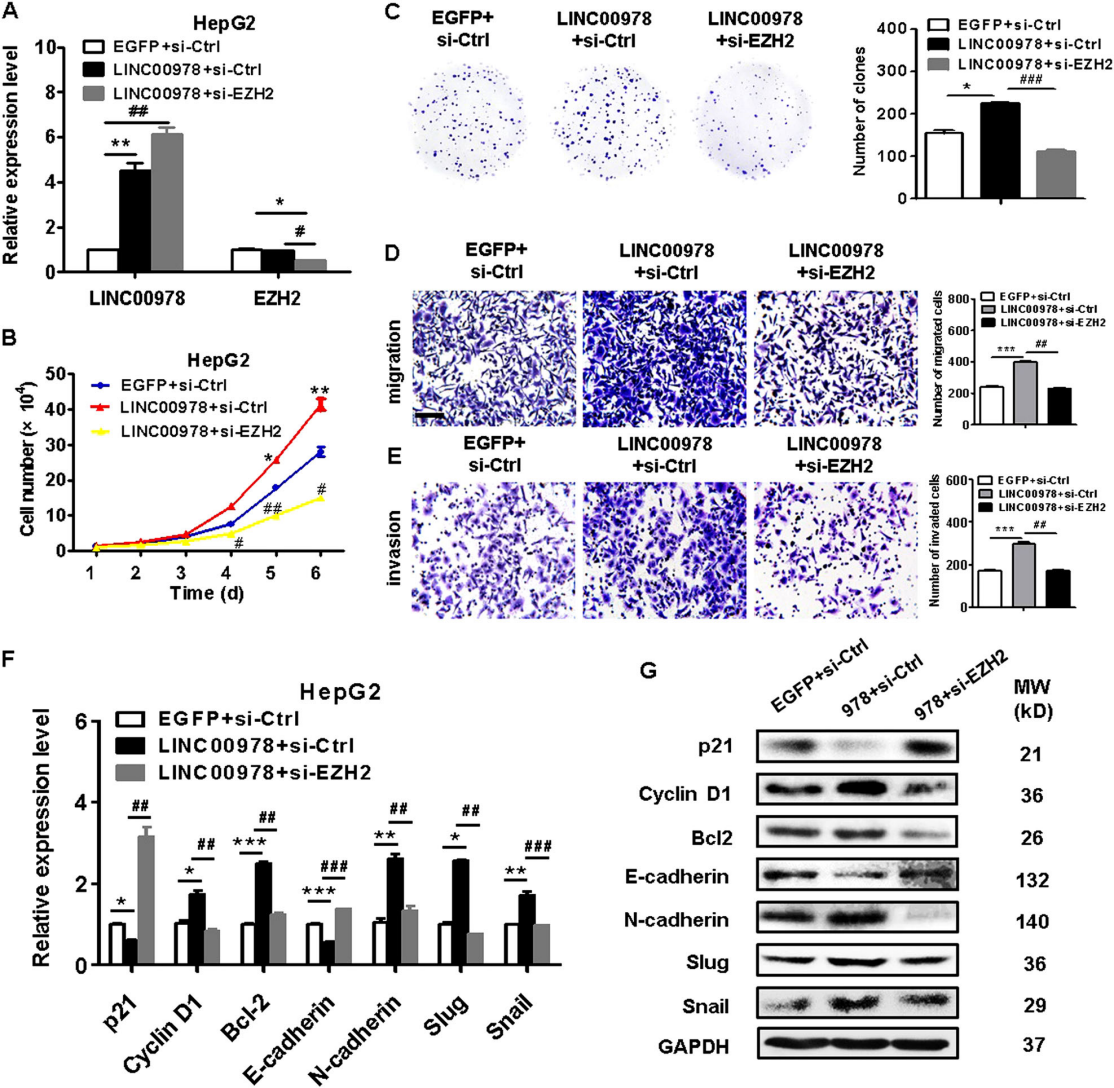
Knockdown of EZH2 reversed the oncogenic roles of LINC00978 in HCC cells. **a** Relative expression levels of LINC00978 and EZH2 in HepG2 cells transfected with LINC00978 alone or LINC00978 + si-EZH2. **b** Growth curves of HCC cells transfected with LINC00978 alone or LINC00978 + si-EZH2. **c** Colony formation assays for HCC cells transfected with LINC00978 alone or LINC00978 + si-EZH2. **d**, **e** Transwell migration (**d**) and matrigel invasion (**e**) assays were performed to investigate the migratory and invasive abilities of HepG2 cells transfected with LINC00978 alone or LINC00978 + si-EZH2. **f** qRT-PCR analyses of proliferation and EMT-specific markers in HepG2 cells transfected with LINC00978 alone or LINC00978 + si-EZH2. Scale bar: 100 μm. *P < 0.05, **P < 0.01, ***P < 0.001, compared with EGFP group; ^#^*P* < 0.05, ^##^*P* < 0.01, ^###^*P* < 0.001, compared with LINC00978 + si-Ctrl group

We next wanted to know the importance of EZH2 to the oncogenic roles of LINC00978 in HCC. We overexpressed LINC00978 and knocked down EZH2 in HCC cells simultaneously (Fig. 5a). The results of cell counting and colony formation assays showed that the proliferation of HepG2 cells with LINC00978 overexpression and EZH2 knockdown was decreased compared with that of HepG2 cells with LINC00978 overexpression alone (Fig. 5b, c). The number of migrated and invaded cells was decreased in co-transfection group compared with LINC00978 overexpression alone group (Fig. 5d, e). In addition, the inhibition of p21 and E-cadherin expression levels by LINC00978 overexpression was reversed by EZH2 knockdown (Fig. 5f, g). Moreover, the increased expression of Cyclin D1, Bcl-2, N-cadherin, slug, and snail by LINC00978 overexpression was reversed by EZH2 knockdown. Collectively, these data indicate that EZH2 is critically involved in the promotion of HCC cell proliferation, migration and invasion by LINC00978.

### LINC00978 exerts oncogenic roles in HCC through the inhibition of p21 and E-cadherin

**Fig. 6:**
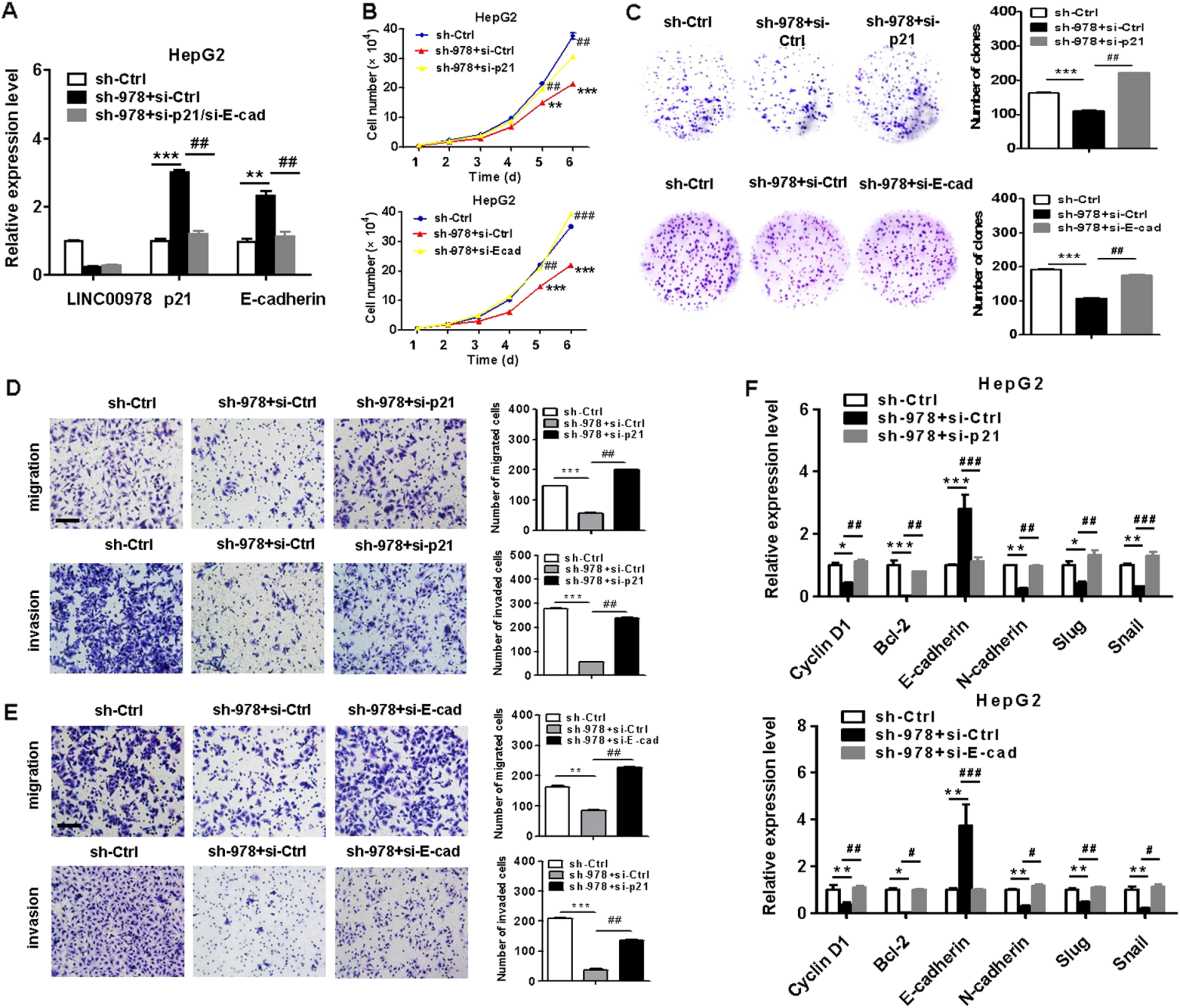
LINC00978 exerts oncogenic roles in HCC cells through the inhibition of p21 and E-cadherin. **a** Relative expression levels of p21 and E-cadherin in HepG2 cells transfected with sh-LINC00978 alone or sh-LINC00978+ si-p21/si-E-cadherin. **b** Growth curves of HCC cells transfected with sh-LINC00978 alone or sh-LINC00978 + si-p21/si-E-cadherin. **c** Colony formation assays for HCC cells transfected with sh-LINC00978 alone or sh-LINC00978 + si-p21/si-E-cadherin. **d**, **e** Transwell migration (**d**) and matrigel invasion (**e**) assays were performed to investigate the migratory and invasive abilities of HepG2 cells transfected with sh-LINC00978 alone or sh-LINC00978 + si-p21/si-E-cadherin. **f** qRT-PCR analyses of proliferation and EMT-specific markers in HepG2 cells transfected with sh-LINC00978 alone or sh-LINC00978 + si-p21/si-E-cadherin. Scale bar: 100 μm. *P < 0.05, **P < 0.01, ***P < 0.001, compared with sh-Ctrl group; ^#^*P* < 0.05, ^##^*P* < 0.01, ^###^*P* < 0.001, compared with sh-978 + si-Ctrl group

To demonstrate the importance of p21 and E-cadherin inhibition in the roles of LINC00978 in HCC, we co-transfected HepG2 cells with LINC00978 shRNAs and p21 siRNAs (or E-cadherin siRNAs). qRT-PCR results showed that co-transfection reduced upregulation of p21 and E-cadherin by LINC00978 depletion (Fig. 6a). In addition, the results of cell counting and colony formation assays indicated that the proliferation of HepG2 cells co-transfected with sh-LINC00978 and si-p21 (or E-cadherin siRNAs) was increased compared with HepG2 cells transfected with sh-LINC00978 alone (Fig. 6b, c). The number of migrated and invaded cells was also increased in co-transfection group compared with sh-LINC00978 alone group (Fig. 6d, e). The expression levels of proliferation and metastasis related genes were also partially reversed in co-transfection group compared with sh-LINC00978 alone group (Fig. 6f). Collectively, these data indicate that LINC00978 promotes HCC cell proliferation, migration and invasion through the downregulation of p21 and E-cadherin.

### LINC00978 knockdown inhibits HCC growth and metastasis

**Fig. 7:**
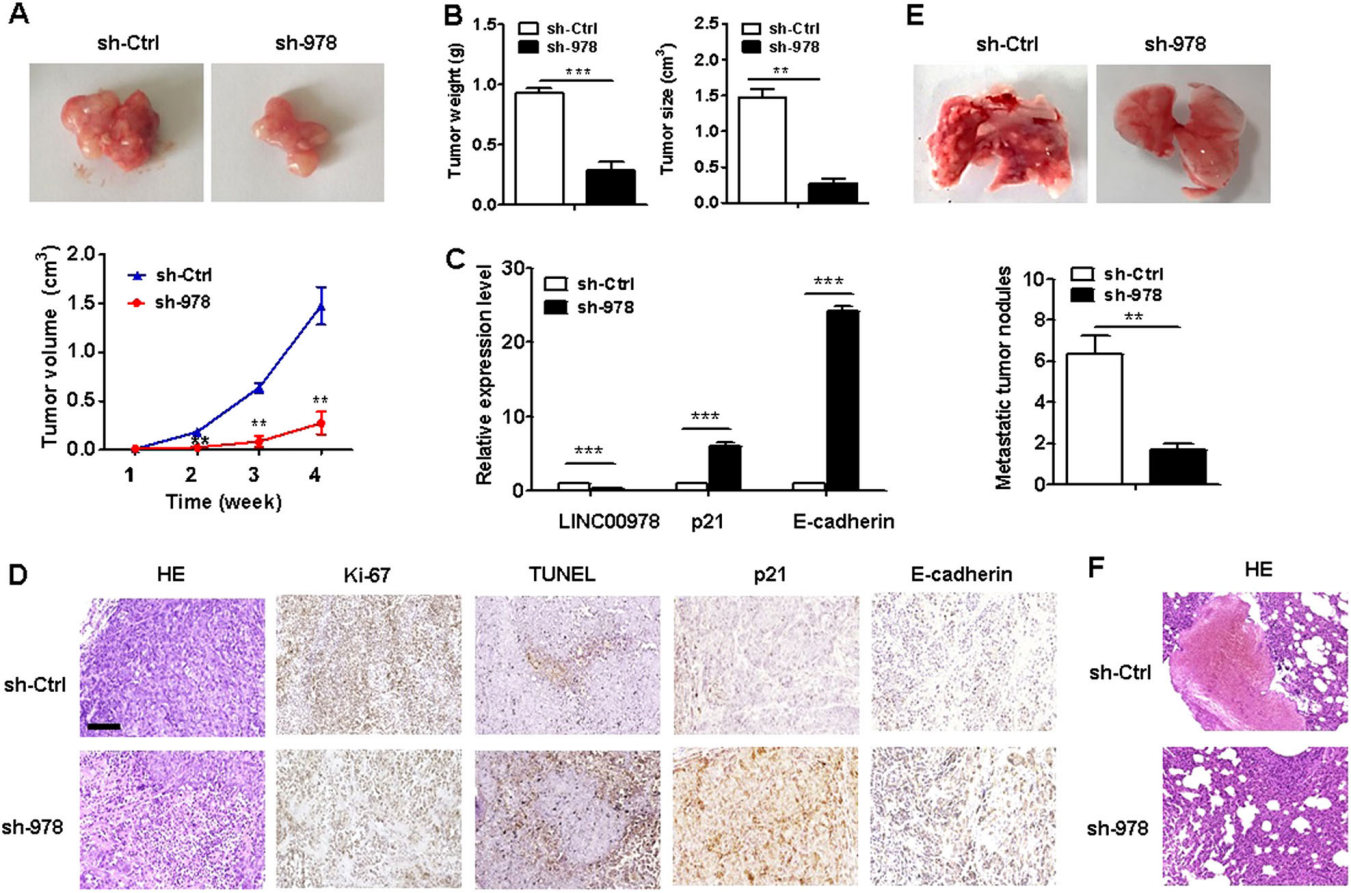
Downregulation of LINC00978 inhibits HCC tumorigenesis in vivo. **a** Tumor growth curves of mice in sh-LINC00978 and sh-control groups. **b** Tumor weights and sizes in mice from sh-LINC00978 and sh-control (sh-Ctrl) groups. **c** qRT-PCR analyses of p21 and E-cadherin expression in tumor tissues of sh-LINC00978 and sh-control groups. **d** Representative images of HE staining, immunohistochemical staining of Ki-67, p21, and E-cadherin, and TUNEL staining of mouse tumor tissues. **e** Metastatic tumor modules in the lungs of mice in sh-LINC00978 and sh-control groups. **f** Representative images of HE staining of mouse lung tissues in sh-LINC00978 and sh-control groups. Scale bar: 100 μm. *P < 0.05, **P < 0.01, ***P < 0.001

To further investigate whether LINC00978 affects HCC progression in vivo, HepG2 cells stably transfected with sh-LINC00978 or sh-Ctrl were inoculated into the nude mice. Four weeks after injection, all the mice developed xenograft tumors at the injection site and tumor growth rate in sh-LINC00978 group was significantly lower than that in sh-Ctrl group (Fig. 7a). Moreover, tumor weight and tumor size in sh-LINC00978 group was significantly lower than that in sh-Ctrl group (Fig. 7b). Next, we used qRT-PCR to detect the expression of LINC00978, p21 and E-cadherin in mouse xenograft tumor tissues. LINC00978 was lower while p21 and E-cadherin was higher in sh-LINC00978 group than that in sh-Ctrl group (Fig. 7c). The results of immunohistochemistry confirmed that tumors from sh-LINC00978 group displayed lower intensity of Ki-67 staining but higher intensity of TUNEL staining than those from sh-Ctrl group (Fig. 7d). Increased levels of p21 and E-cadherin expression were also observed in tumors from sh-LINC00978 group compare to sh-Ctrl group (Fig. 7d). Moreover, we found that mice intravenously injected with sh-LINC00978 HepG2 cells developed more metastatic nodules in the lungs than that injected with sh-Ctrl HepG2 cells at 6 weeks after inoculation (Fig. 7e, f). These results indicate that LINC00978 promotes the growth and metastasis of HCC through suppression of p21 and E-cadherin.

## Discussion

Previous studies suggest that lncRNAs are involved in the development and progression of HCC^20–23^. LINC00978 was first identified in a microarray analysis as one of the mostly altered lncRNAs by resveratrol in lung cancer cells^24^. LINC00978 can be used as a potential biomarker in patients with non-small cell lung cancer^15,16^, breast cancer^17^, and gastric cancer^18,19^. However, the roles, mechanisms and implications of LINC00978 in HCC remain unclear.

Circulating lncRNAs provide a blood-based biomarker for cancer detection^25^. For instance, lnc-ATB is upregulated in HCC and high level of lnc-ATB is associated with poor prognosis^26^. In this study, we first identified LINC00978 as an overexpressed lncRNA in HCC by analyzing GEO microarray datasets. We further validated this finding in a cohort of tumor and non-tumor tissues. Moreover, we found that LINC00978 was upregulated in serum of HCC patients and its upregulation could discriminate HCC patients from liver benign disease patients and healthy controls, suggesting that LINC00978 may serve as a potential diagnostic and prognostic biomarker for HCC. We further explored the biological roles of LINC00978 in HCC by using shRNA/plasmid-mediated gene silencing/overexpression. We showed that LINC00978 knockdown inhibited while LINC00978 overexpression promoted HCC cell proliferation, migration, and invasion. In support of the in vitro findings, LINC00978 knockdown also retarded HCC growth and metastasis in vivo, indicating that LINC00978 is critical for HCC progression.

LncRNAs regulate HCC progression through distinct mechanisms. LINC01554 suppresses HCC via downregulating PKM2 expression and inhibiting Akt/mTOR signaling pathway^27^. The long non-coding RNA cancer susceptibility 9 binds to heterogeneous nuclear ribonucleoprotein L to regulate AKT signaling^28^. HOXD-AS1 facilitates HCC metastasis by acting as miRNA sponge for miR-130a-3p to upregulate Sox4 expression^29^ and lncSox4 promotes the self-renewal ability of HCC cells through STAT3-mediated Sox4 upregulation^30^. MALAT1 induces the expression of splicing factor SRSF1, which enhances the production of antiapoptotic splicing isoforms and activates the mTOR pathway by modulating the alternative splicing of S6K1^31^. LncBRM associates with BRM to initiate the BRG1/BRM switch and the BRG1-embedded BAF complex and triggers activation of YAP1 signaling, which maintains the self-renewal of liver cancer stem cells (CSCs) and promotes tumor initiation^32^. In this study, we identified p21 and E-cadherin as targets of LINC00978 in HCC cells. LncRNAs can regulate gene expression at different levels, including chromatin modification, transcription, post-transcription, and translation. To confirm the regulatory mechanism of LINC00978, we performed RNA immunoprecipitation assays and ChIP assays and found that p21 and E-cadherin could be regulated through histone modification by LINC00978-mediated EZH2 recruitment. EZH2 is a key component of polycomb repressive complex 2 and functions as a histone H3K27 trimethyltransferase. EZH2 is frequently overexpressed in a variety of cancer including HCC and targeting EZH2 has shown promising therapeutic effects in cancer^33,34^. Previous studies demonstrate that EZH2 binding and recruitment is a common mechanism for lncRNAs-mediated gene silencing^2^. We confirmed that EZH2 knockdown reversed the oncogenic roles of LINC00978 in HCC, suggesting that EZH2 recruitment and epigenetic silencing of downstream genes is a novel mechanism for the roles of LINC00978 in cancer.

p21, a cyclin-dependent kinase (CDK) inhibitor, is downregulated in a variety of cancers and plays critical roles in multiple cellular processes by directly binding to kinases related to G1/S transition. EMT is a progressive biological process in which epithelial cells gradually acquire a mesenchymal-like phenotype. E-cadherin is a core marker of epithelial cells and be a key molecule in the EMT process. Wei et al. demonstrate that SOX21-AS1 epigenetically silences p21 via recruiting EZH2 to its promoter, which promotes HCC cell proliferation and metastasis^35^. Zhou et al. show that SPRY4-IT1 interacts with EZH2 and epigenetically represses E-cadherin expression to promote HCC cell proliferation and invasion^36^. Here, we found p21 and E-cadherin were downstream targets involved in LINC00978-mediated promotion of HCC cell proliferation, migration and invasion. Rescue experiments indicate that inhibition of p21/E-cadherin potentially reverses the anti-tumor activities of LINC00978 knockdown. These data indicate that LINC00978 contributes to HCC progression partly through the epigenetic silencing of p21/E-cadherin expression by binding to EZH2. However, other possible targets and mechanisms need to be further investigated.

Collectively, we demonstrated that LINC00978 was upregulated in tumor tissues and serum of HCC patients. LINC00978 promoted HCC cell proliferation, migration and invasion by favoring cell cycle progression, inhibiting cell apoptosis and inducing EMT. LINC00978 functions as a negative regulator of p21 and E-cadherin by binding to EZH2. Our study provides new insight into the oncogenic roles of LINC00978 in HCC progression, therefore, which may provide a novel diagnostic marker and therapeutic target for HCC.
